# Dynamics between Cancer Cell Subpopulations Reveals a Model Coordinating with Both Hierarchical and Stochastic Concepts

**DOI:** 10.1371/journal.pone.0084654

**Published:** 2014-01-09

**Authors:** Weikang Wang, Yi Quan, Qibin Fu, Yu Liu, Ying Liang, Jingwen Wu, Gen Yang, Chunxiong Luo, Qi Ouyang, Yugang Wang

**Affiliations:** 1 State Key Laboratory of Nuclear Physics and Technology, School of Physics, Peking University, Beijing, P. R. China; 2 Center for Microfluidic and Nanotechnology, The State Key Laboratory for Artificial Microstructures and Mesoscopic Physics, School of Physics, Peking University, Beijing, P. R. China; Tokai University, Japan

## Abstract

Tumors are often heterogeneous in which tumor cells of different phenotypes have distinct properties. For scientific and clinical interests, it is of fundamental importance to understand their properties and the dynamic variations among different phenotypes, specifically under radio- and/or chemo-therapy. Currently there are two controversial models describing tumor heterogeneity, the cancer stem cell (CSC) model and the stochastic model. To clarify the controversy, we measured probabilities of different division types and transitions of cells via *in situ* immunofluorescence. Based on the experiment data, we constructed a model that combines the CSC with the stochastic concepts, showing the existence of both distinctive CSC subpopulations and the stochastic transitions from NSCCs to CSCs. The results showed that the dynamic variations between CSCs and non-stem cancer cells (NSCCs) can be simulated with the model. Further studies also showed that the model can be used to describe the dynamics of the two subpopulations after radiation treatment. More importantly, analysis demonstrated that the experimental detectable equilibrium CSC proportion can be achieved only when the stochastic transitions from NSCCs to CSCs occur, indicating that tumor heterogeneity may exist in a model coordinating with both the CSC and the stochastic concepts. The mathematic model based on experimental parameters may contribute to a better understanding of the tumor heterogeneity, and provide references on the dynamics of CSC subpopulation during radiotherapy.

## Introduction

Tumors are often heterogeneous in which individual tumor cells exist in different phenotypes with distinct functional properties [1]. Clinically, tumors from different patients, whether leukemic or solid, often exhibit significant heterogeneity in terms of morphology, cell surface markers, genetic lesions, cell proliferation kinetics, and response to therapy [2]. Therefore, it is of fundamental importance to understand the molecular and cellular basis of the heterogeneity. Currently there are two controversial models describing the heterogeneity in tumor, the CSC model and the stochastic model. The CSC model, also known as the hierarchy model, suggests that the growth and progression of many cancers are driven by small but distinctive subpopulations of CSCs, and the tumor is a caricature of normal tissue development where stem cells maintain normal tissue hierarchies [3]. The CSCs at the apex of hierarchical structure can not only maintain themselves by self-renewal, but also differentiate into NSCCs. In contrast, the stochastic model, also known as clonal evolution model, predicts that a tumor is biologically homogeneous and the behavior of the cancer cells is randomly influenced by unpredicted intrinsic and/or extrinsic factors [3].

The two models evoked great interests in both experimental and theoretical studies. In experimental studies, although the mechanism of the tumor heterogeneity is still unclear, there is strong evidence that cancer is a cellular hierarchy with CSCs at the apex [2], [4]–[7], indicating that cancer therapy may require elimination of CSCs [4], [8]. These papers supported the CSC model and evoked novel strategies on targeting CSCs to treat cancer [2], [4]–[7]. However, several other papers showed that the phenotypic plasticity within tumors may produce bidirectional inter-conversion between CSCs and NSCCs, resulting in dynamic variation in the relative abundance of CSCs [1], [9]–[11]. Vesuna *et al* found that transient expression of *Twist* can induce the stem cell phenotype in multiple breast cell lines and that decreasing *Twist* expression partially reverses the stem cell molecular signature[12]. Morel *et al* reported that breast CSCs can be generated through EMT cascade [13]. Liang *et al* suggested that CSCs are inducible by increasing genomic instability in cancer cells [14]. Interestingly, Chaffer *et al* reported that normal and neoplastic non-stem cells can spontaneously convert to a stem-like state [9]. More importantly, Iliopoulos *et al* reported that breast CSCs can be induced from NSCCs via IL6 secretion and the two cell populations can reach dynamic equilibrium [1]. Recently, Gupta *et al* described a model that phenotypic equilibrium in populations of cancer cells is achieved via stochastic state transitions [10]. Our previous studies also showed the *in situ* transitions and phenotype dynamic equilibrium between CSCs and NSCCs, either with or without radiation treatment [11].

In theoretic studies, hot debate also has been stimulated among different papers. Beretta *et al* analyzed asymptotic behavior of CSC proportion and the case when there are no transitions from non-stem to stem cell [15], showing the stability of CSCs percentage in a mathematical way. Gupta *et al* developed a Markov model to explain the phenomenon that a purified phenotype subpopulation finally returns to equilibrium phenotypic proportions under the condition that cells transit stochastically among different states [10]. This model predicts that non-stem cells like basal and luminal have a non-zero probability to become stem-like state. Zapperi *et al* analyzed kinds of mathematical models and proposed that imperfect sorting could be an alternative explanation for the “purified” subpopulation returning to equilibrium proportions [16].

The CSC model and the stochastic model suggest different clinical strategies of tumor therapy. Presently, the urgency lies in how to improve both models to gain a better understanding of tumor heterogeneity and the dynamic variations of different subpopulations, specifically the CSCs and NSCCs in tumor. We constructed a mathematic model based on parameters measured from experiments, specifically the types and rates of divisions and transitions. The results showed that the experimental dynamics between CSC and NSCC subpopulations can be simulated via the model, either with or without radiation treatment. Further analysis demonstrated that the experimental detectable equilibrium CSC proportion can be achieved only when the stochastic transitions from NSCCs to CSCs occur, suggesting tumor heterogeneity may exist in a model coordinating with both the CSC and the stochastic concepts.

## Equations and Assumptions

**Figure 1:**
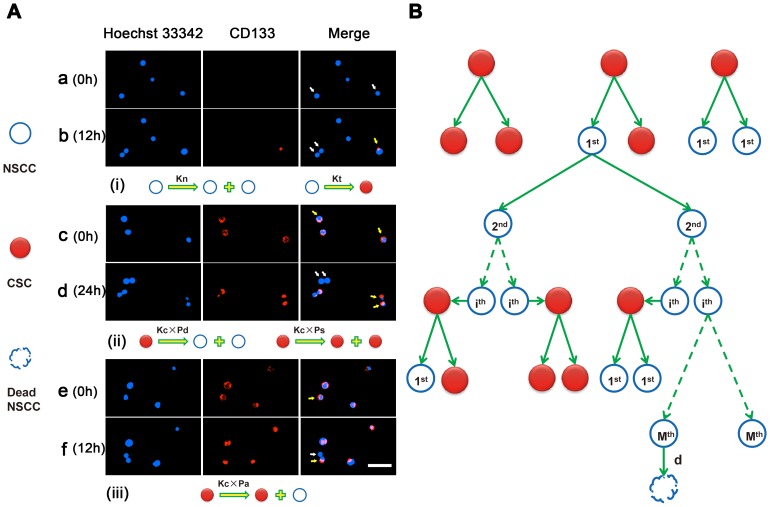
Divisions and transitions of CSCs/NSCCs as well as kinetics and scheme of the model. (A). Typical division types of CSCs/NSCCs and transition from NSCC to CSC. Scale bar equals 50 µm. (a–b) Typical *in situ* division type of a NSCC (white arrow) and transition from a NSCC to a CSC (yellow arrow); (c–d). Typical *in situ* symmetric divisions of CSCs: self-renewal (one CSC divides into two CSCs) and differentiation (One CSC divides into two NSCCs); NSCC (white arrow), CSC (yellow arrow). (e–f). Typical *in situ* asymmetric division of CSC (One CSC divide into one CSC and one NSCC); NSCC (white arrow), CSC (yellow arrow). (i). Kinetic equations that correspond to the phenomena in a and b. (ii) Kinetic equations that correspond to the phenomena in c and d. (iii)Kinetic equation that corresponds to the phenomenon in e and f. (B). Scheme of the model based on the experimental results.

Previous studies suggested that CD133-positive cells are the potential CSCs subpopulation in SW620 human colon cells [11], [17], [18]. By means of *in situ* immunofluorescence, the division types of CSCs and NSCCs through surface marker changes were assayed. For CSCs, both symmetric and asymmetric divisions were captured. That is, a CSC can divide into two CSCs (self-renewal), two NSCCs (differentiation) as well as a CSC and a NSCC (asymmetric division) (Fig. 1A). For NSCCs, only the symmetric division type (proliferation) was captured, that is, a NSCC divides into two NSCCs. Importantly, there are distinctive phenotype transitions from NSCC to CSC independent of cell mitosis (Fig.1A).

Major assumptions:

1. There are CSCs and NSCCs subpopulations in SW620 human colon cancer cells [11].
2. A CSC can divide symmetrically into two CSCs (self-renewal) or two NSCCs (differentiation) with probability *P_S_* or *P_D_* respectively (Fig. 1A). In addition, a CSC can divide asymmetrically into a CSC and a NSCC with probability *P_A_* (*P_A_* = 1−*P_S_*−*P_D_*) (Fig.1A). Different CSC division types have the same mitosis speed denoted by *K_C_*.
3. A NSCC can divide into two NSCCs (proliferation) with rate of *K_N_* (Fig.1A).
4. A NSCC can convert into a CSC with rate of *K_T_* (Fig. 1A) [11].
5. NSCC has limited proliferate potential and could go through senescence with lifespan of *M* generation [19], [20]. The *M_th_* generation dies with a rate of *d* (Fig. 1B). The value of *d* and *M* are simply set to be 1 and 50 as suggested previously [21].

The schematic of the model is shown in Fig. 1B. According to the assumptions listed above, the dynamics between CSCs and NSCCs can be described with ordinary differential equations (ODEs) (Equation (1)). In ODEs, we find that *P_S_, P_D_* and *P_A_* appear in certain combinations. So these three parameters could be incorporated into one parameter 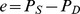. 
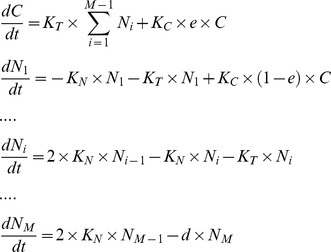
(1)


*C* denotes the number of CSCs and *N_i_* denote the number of NSCCs; *i = 1, 2,* …, *M*.

It is well known that radiation treatment can cause a lot of damages in cells, among which DNA double strand breaks (DSBs) are the most toxic [22]. Here we add death rates correlated with DSBs dynamics into our model when cells were irradiated. After radiation, the number of DSBs quickly increased and saturated in the irradiated cells [23], then decreased due to DNA repair. Therefore, based on DSBs' dynamics [24], [25], the death rate could be described as
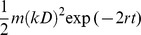

*k* denotes DSB's production on average per unit dose. *D* denotes dose. *r* is repair rate of DSBs, *r_C_* and *r_N_* represent repair rate of CSC and NSCC respectively. *m* stands for lethal mis-repair rate of per DSB pair. In present model, *m_C_* and *m_N_* represent lethal mis-repair rates of CSC and NSCC respectively (Details can be found in Equations S1 in File S1).

## Results

### Parameters measured via in situ experiments

The probabilities of CSCs' division types and percentage of transition of NSCCs were determined using *in situ* immunofluorescence (Fig. 1). To be consistent with experiment results of population dynamics, we estimated *K_T_*, *K_N_* and *K_C_* by calculating the quantity change of sorted CSCs and NSCCs and percentage of NSCCs' transition in one day. Because CSCs and NSCCs' cell cycles are both approximately one day, the division of newly born NSCCs in sorted CSCs population contributes little to quantity change in one day and the division of new CSCs in sorted NSCCs is not significant (Equations used in the estimation are shown in Equations S2 in File S1). The values of these parameters are shown in Table 1.

**Table 1:** Parameters collected from *in situ* experiments.

Parameter	Symbol	value
self-renewal probability of a CSC (a CSC divides into two CSCs)	*P_S_*	0.777±0.075
asymmetric division probability of a CSC (a CSC divides into one CSC and one NSCC)	*P_A_*	0.164±0.060
differentiation probability of a CSC (a CSC divides into two NSCCs)	*P_D_*	0.059±0.017
the rate of a NSCC converts into a CSC (day^−1^)	*K_T_*	0.269
proliferation rate of a NSCC (day^−1^) (the rate of a NSCC divides into two NSCCs)	*K_N_*	0.659
CSC mitosis speed (day^−1^)	*K_C_*	0.849

After radiation treatment, the average of DSB's production in a cell is reported to be 25–35/Gy [26]. And *r* is calculated from half-life of DSBs or foci and its order of magnitude is ∼10/day [23], [27]. Since CSC has higher ability to repair DNA damage [28], the assumption that *r_C_*>*r_N_* is made. Here we set *r_N_* = 10 and *r_C_* = 15. The survival fractions (*S*) of CSCs (*S_C_*) and NSCCs (*S_N_*) under 2 Gy radiation treatment are measured from the experiments. Therefore, the lethal mis-repair rate of CSCs (*m_C_*) and NSCCs (*m_N_*) can be calculated by following equation (Equation (2)),
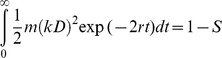
(2)


As shown in Table 2, the value of *k*, D, *r_N_*, *r_C_*, *S_C_*, *S_N_*, *m_C_* and *m_N_* are 25, 2, 10, 15, 95.0%, 43.0%, 0.0012 and 0.0092 respectively.

**Table 2:** Parameters under irradiation surroundings.

Parameter	Symbol	value
DSBs' production on average per unit dose (Gy^−1^)	*k*	25
Dose of irradiation (Gy)	D	2
DSBs repair rate of NSCC (day^−1^)	*r_N_*	10
DSBs repair rate of CSC (day^−1^)	*r_C_*	15
survival fraction of CSCs after 2Gy irradiation	*S_C_*	95.0%
survival fraction of NSCCs after 2Gy irradiation	*S_N_*	43.0%
lethal mis-repair rate of CSC (day^−1^)	*m_C_*	0.0012
lethal mis-repair rate of NSCC (day^−1^)	*m_N_*	0.0092

### Simulation of long-term dynamic variations between CSC and NSCC subpopulations

Based on the parameters, we then analyzed the dynamics of the CSC proportion (define as 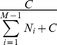) under different initial conditions via the mathematical model (simulation of cell number variation is shown in Table S1 in File S1 and Fig. S1).

**Figure 2:**
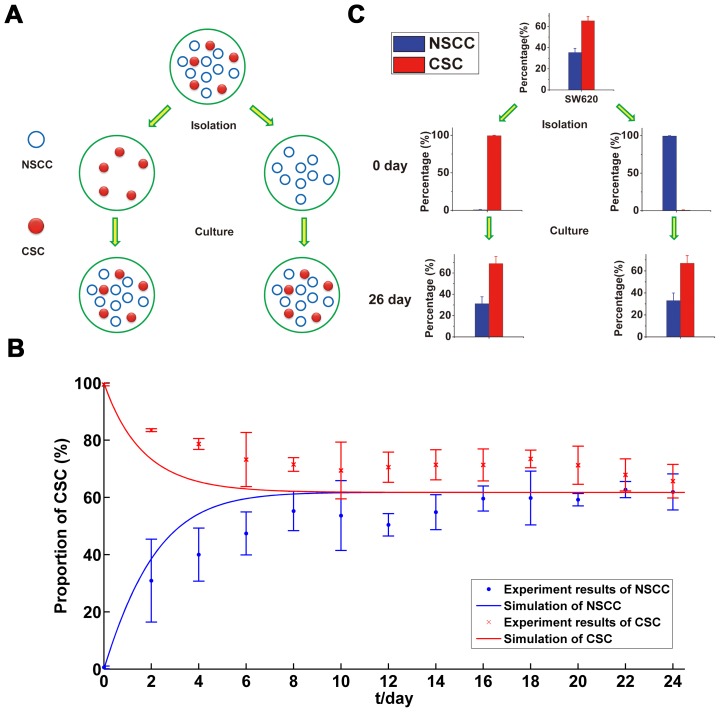
Experiments procedures, results and the simulations of long-term dynamics between CSC and NSCC subpopulations. (A). Diagram of experiment procedures; (B). Comparison between simulation results and experiment results; (C). Experimental results of long-term equilibrium CSC proportions from initial purified CSCs and NSCCs.

Theoretically, it is shown that the CSC proportion finally reaches a steady value no matter what the initial condition is (Fig. 2A). Comparing simulation results with experimental data reported previously [11], it is clear that the steady value computed by this model is close to the experimental results(Fig. 2B), demonstrating parameters getting from the *in situ* immunofluorescence can predict the tendency of the dynamics between CSCs and NSCCs subpopulation(experiment data are shown in Tables S2–S3 in File S1). In addition, purified NSCCs and CSCs sorted from SW620 cell line by FACS were cultured, and the CSC proportions at day 26 post inoculation were tested. As shown in Fig. 2C, CSC proportions of different initial cultures reach the same steady value which equals the CSC proportion in unsorted SW620 cells.

### Parameter sensitivity analysis

The responses of CSC proportion to the change of parameters at equilibrium are analyzed (parameters are shown in Table 1). Regularly, each parameter is increased or decreased by one percent and the change of the CSC proportion at equilibrium is calculated as reported previously [29]. *M* is an integer, so the change of *M* is plus or minus 1. As shown in Fig. S2, when *K_T_*, *K_N_*, *K_C_* and *e* are increased by 1 percent, the CSC proportion at the equilibrium would increase 0.3%, decrease 0.5%, increase 0.2% and increase 1.1% respectively. Among the parameters, *M* is an insensitive parameter, which is set to be 50 as suggested previously [21]. According to calculation, *M* is an insensitive parameter in a large range. So the choice of M's value places little influence on simulation of equilibrium. Other sensitive parameters including *e* (*e* = *P_S_*−*P_D_*), *K_N_*, *K_T_* and *K_C_* are all measured in experiments

### Test the parameters and the dynamics between CSC and NSCC subpopulations via cellular automaton method

To further validate the parameters and the dynamics between CSCs and NSCCs, we then studied the dynamics between CSC and NSCC subpopulations with the parameters via cellular automaton method. Cellular automaton is based on behavior of individual cell and interaction between individuals. It is widely used for modeling multi-cellular biological systems including tumor. It could reflect the discrete property of tumor which is neglected in the ODE method[30]. By using cell automaton method, a better understanding of how tumor grows in microscopic scale can be obtained[31]. As the concept of CSC comes out, cellular automaton method is used for simulation of CSCs [32]–[36].

**Figure 3:**
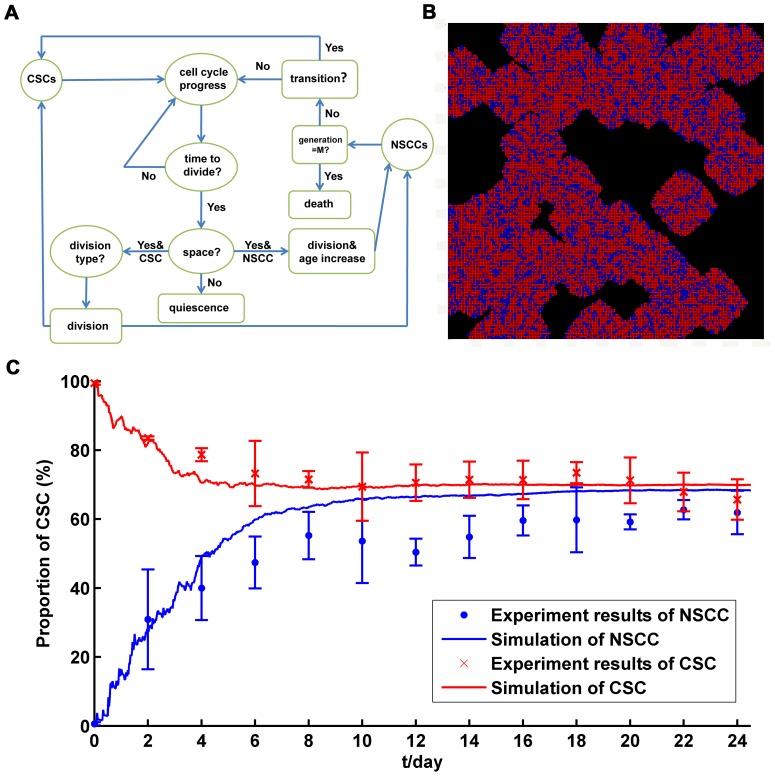
The long-term dynamics between CSC and NSCC subpopulations via cellular automaton simulation method. (A). Calculation scheme for cellular automaton method. (B). Typical result of simulation with cellular automaton method (initial condition is that all the cells are NSCCs). Red: CSC; Blue: NSCC; Black: vacant lattice. (C). Comparison between simulation results with cellular automaton method and experimental results.

The calculation scheme can be found in Fig.3A. In each time step, A NSCC decides whether to die or whether to transform into a CSC. NSCCs and CSCs progress one step in their cell cycles respectively. A cell will divide into two cells when it finishes one cell cycle. If there is no vacant site for the cell to divide, it would become quiescent. If there is space for the cell to divide, for a CSC, it would decide division type by chance; for a NSCC, it would divide and both the daughter cells' generations increase by 1.

As shown in Fig.3C, with the parameters, the simulation shows consistency with the experiment data and the CSC proportion from each group also reached the steady value, further indicating those parameters collected from the experiments are reliable. In addition, the results of cellular automaton method provide more detailed information of the dynamics. During the proliferation, CSCs and NSCCs may firstly form colonies, and then expand around (Video S1-2). Finally, CSCs and NSCCs scattered uniformly throughout the whole area. It is possible that all off-springs of a CSC or a NSCC are CSCs and NSCCs for several generations. If these CSCs or NSCCs connect with other CSCs or NSCCs respectively, they become aggregations in certain areas (Fig.3B).

### Simulation of long-term dynamic variations between CSC and NSCC subpopulations after radiation treatment

**Figure 4:**
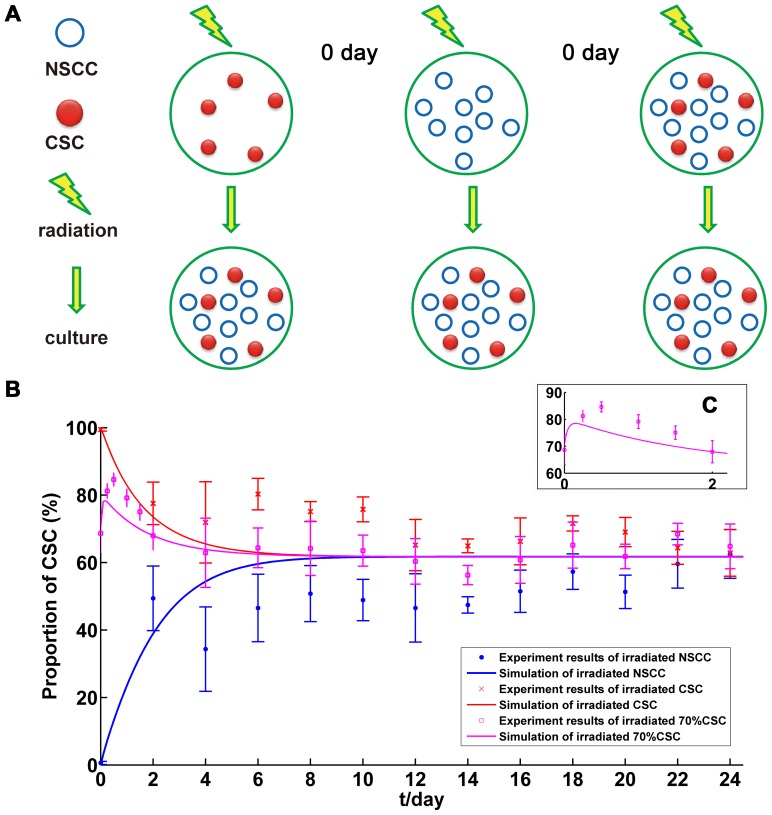
Radiation experiments procedures and simulations of long-term dynamics between CSC and NSCC subpopulations. (A). Diagram of experiment procedures with radiation treatment; (B). Comparison between simulation and experiment results in 0–24 day (radiation is applied when t is 0 day); (C). Amplified image of the results from irradiated 70% CSC group (0–2d) (radiation is applied when t is 0 day).

The dynamic variations between CSCs and NSCCs after radiation treatment are simulated with several additional parameters were then performed (Table 2) (simulation of cell number variation is shown in Table S4 in File S1and Fig. S3). The results showed that the model simulation gives an acceptable prediction on experimental results as we previously reported [11]. As shown in Fig. 4B, CSC proportions of all groups from different initial proportions can finally reach the same steady value as the cases without radiation, indicating short term radiation cannot disturb the long term dynamic equilibrium between the CSCs and NSCCs. Interestingly, in the mixture of 70% CSCs and 30% NSCCs group, simulation shows that CSC proportion rises in the beginning quickly and falls down in two days (Fig. 4C). This is also in accordance with the experimental results as we reported previously [11].

### Imperfect sorting cannot explain the dynamic equilibrium between NSCCs and CSCs

The dynamic equilibrium between CSCs and NSCCs is an interesting phenomenon [1], [9]–[11]. This phenomenon, which is recently reported by several papers, may have profound impacts on the understanding of tumor heterogeneity as well as clinical therapy strategies[10]. Analysis of the phenomenon also showed that, a stable equilibrium CSC proportion between 0 and 1 is easily to achieve if there exist transitions from NSCCs to CSCs (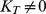). If *K_T_* equals 0, the non-zero equilibrium CSC proportion exists only under the condition that 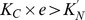, that is, the net proliferation rate of CSCs is higher than that of NSCCs (details can be found in Discussion S1 in File S1 and Fig. S4), which is also not the case in our experiments and other reports [2], [37].

**Figure 5:**
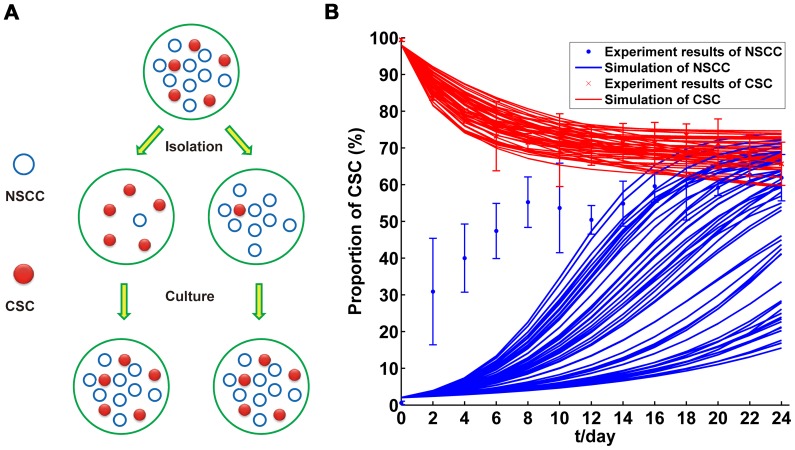
Impact on the long-term dynamics between CSC and NSCC subpopulations with sorting error. (A). Diagram of sorting error in the experiments; (B). Comparison between simulation (*K_T_* = 0) and experiment results.

An alternative explanation for dynamic equilibrium suggested by Zapperi *et al* is that this phenomena might due to the imperfect sorting of the cells via flow cytometry instead of the transitions from NSCCs to CSCs. The imperfect sorting is unavoidable in the experiments, resulting in some cells into the wrong group as a minority (Fig.5A). As shown in Discussion S1 in File S1, 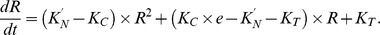



*R* is the CSC proportion in the whole population.

If *K_T_* is 0, 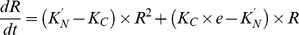. Under the situation of imperfect sorting, *R* is very low in sorted NSCCs at the beginning. So 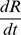 nearly equals zero. So the increase of *R* will be insignificant in the first several days. According to our experiment data, 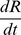 is larger than 0.1 in the first two days. If *R* is 0.02 at the beginning, 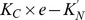 should be larger than 5. This is against experiment records on cell proliferation. However, if *K_T_* is not 0, 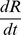 is approximately equal to *K_T_* at the beginning. The increase of *R* will be more close to our experiment data.

To better illustrate this probability, we analyzed theoretically in our model with sorting error of CSCs and NSCCs as *θ* percent (normally *θ*≤2 according to the instructions of the flow cytometry). If there are no transitions from NSCCs to CSCs (*K_T_* = 0), the model cannot fit experimental data of CSC proportion dynamics gained from experiments with *θ*. Simulated annealing algorithm is used to fit our experiment data whose initial condition is “purified” CSCs, because this process could be achieved with *K_T_* = 0. Then we get 50 parameter combinations of *K_C_*, *K_N_* and *e*. As shown in Fig.5B, the results showed that, although minority CSCs will lead the population to stable equilibrium CSC proportion and these parameters fit the experiment data of purified CSCs precisely, none of these parameter combinations could fit experiment data of purified NSCCs well. As shown in Fig.5B, the differences between simulation and experiment results lie in time span for reaching the equilibrium. This value is largely dependent on *M*. Thus, to get the curve that is similar with experiment data, *M* should be around 5 or less. This is obviously against the experimental data[21], [38]. Therefore, imperfect sorting cannot explain the dynamic equilibrium between CSCs and NSCCs.

## Discussion

Tumor heterogeneity indicates important implications for successful cancer therapies [2]. Currently there are two models describing the heterogeneity in tumor, the stochastic and CSC models. The essential difference between them is that every cell or just a distinct subset tumor cells have the potential to behave like a CSC [2]. To clarify the two concepts, we started from the CSC concept with sorting the CSC and NSCC subpopulations and culturing them separately. Then we measured the probabilities of CSCs' division types and transitions of NSCCs via *in situ* immunofluorescence as described previously [11], [39], [40]. Based on the parameters measured from the experiments (Fig. 1 and Table 1), we constructed a mathematic model coordinating with both CSC and stochastic concepts. The results showed that the model can simulate the tendency of experimental dynamics of NSCC and CSC subpopulations, either with or without radiation treatment (Fig.2 and Fig. 4).

The stochastic model predicts that a tumor is biologically homogeneous and the behavior of the cancer cells is randomly influenced by unpredicted intrinsic and/or extrinsic factors[3]. However, there were increasing evidences supported the existence of CSCs in the past two decades[4]. Traditionally, stochastic models usually define several mutation phenotypes in tumor and the transition rates between these phenotypes. These transitions are usually unidirectional, from the benign types to invasive types [41], [42]. However, our *in situ* experimental results showed that the transitions between CSCs and NSCCs are definitely not unidirectional (Fig.1A), In contrast, NSCCs can transit into CSCs independent of cell mitosis and, CSCs can generate NSCCs via differentiation as well as asymmetric division dependent of cell mitosis (Fig.1A). In addition, genetic instability is one of the most important rules in stochastic model. Through accumulated genetic or epigenetic changes, susceptible phenotype could become resistant phenotype. In the perspective of colony, tumor evolves to become more resistant to therapy [42].

Nevertheless, the advent of CSCs reveals that CSCs is the engine of tumor growth and the resistance to standard chemo- and radio- therapy [5]–[7], [43], showing a more organized hierarchical structure than that indicated by stochastic model. The CSC model suggests that the growth and progression of tumors are driven by small but distinctive subpopulations of CSCs [3]. However, several recent papers and current experiments clearly showed the existence of the *de novo* generation of CSCs from NSCCs (Fig.1) [1], [9]–[11]. The transitions from NSCCs to CSCs indicated that CSC model is not enough to explain the tumor heterogeneity and, essentially supported the concept of stochastic model. Theoretically, if there are no transitions from NSCCs to CSCs (*K_T_* = 0), our model is just the case of CSC model. However, under the initial condition of purified NSCCs, if the transitions do not existed, the CSC proportion in the culture will always be zero. This is obvious not the case observed in our experiments as well as several other reports (Fig.2B and Fig.3C) [1], [9]–[11]. Therefore, CSC model could not explain the phenomena observed in experiments. As shown in the part of results (Fig. 5), imperfect sorting cannot make up this flaw of CSC model.

It is interesting that we started from the CSC model but got the results with features of both the CSC and stochastic concepts (Fig.1A), showing existence of both distinctive CSC/NSCC subpopulations and the stochastic transitions from NSCCs to CSCs.

## Materials and Methods

### Cell culture

Human colon cancer SW620 cells were purchased from Cell Resource Center (IBMS, CAMS/PUMC, Beijing, China) characterized by STR Profiling. Cells were cultured in Dulbecco's modified Eagle's medium, supplemented with 10% fetal bovine serum, 100 units/ml penicillin, and 100 µg/ml streptomycin at 37 °C in 5% CO_2_.

### Cell staining and flow cytometry

Matched subpopulations were separated as described previously [39], [40]. In brief, cells were stained at a concentration of 10^7^ cells per 100 µl of buffer. Anti-CD133/1(AC133)-PE (MiltenyiBiotec) antibody was used for flow cytometric sorting/assay. For all experiments, samples were sorted on a BD FACS Aria II and analyzed on a BD LSR II flow cytometer using BD FACS Diva Software (BD Bioscience). Side scatter and forward scatter profiles were used to eliminate debris and cell doublets.

### *In situ* immunofluorescence

Details of *in situ* immunofluorescence of and chip design are shown in our previous paper [11]. In brief, purified NSCCs and CSCs were stained with the mouse monoclonal antibody against human CD133 antigen coupled with R-phycoerythrin (CD133/1(AC133)-PE from Miltenyi Biotec) together with the DNA-binding dye Hoechst 33342 respectively. After degassing the chip, 25 ml cell (CSCs or NSCCs) suspension were pipetted into the reservoir. The cell suspension was aspired into the cell culture rooms because of the negative pressure. After loading of the sample, the cells in the reservoir were removed and 35 ml of medium was added and cultured normally. After 2 h incubation, cells were photographed for the first time as described below. For immunofluroscence staining of cells at defined time points such as 12 h or 24 h, media in reservoir was removed and 20 ml medium with appropriate concentration of CD133/1(AC133)-PE (Miltenyi Biotec) was added. After incubation, the medium with CD133/1(AC133)-PE in reservoir was removed and 35 ml fresh medium was added and incubated in dark for washing the cells. Cells were washed twice and were immediately photographed.

### Simulated annealing algorithm

Simulated annealing algorithm is a Monte-Carlo algorithm that is often used for optimization problems. The initial parameters are generated randomly and the candidate parameters are also generated randomly by certain rules. These parameters were then used to solve Equation (1). In simulated annealing, we temporarily accept a worse combination of parameters with chance to decrease the risk of local optimization. As temperature falls down, near global optimal solutions would be derived [44]. In fitting process, a parameter combination is accepted ultimately if *∑(simulation-data)^2^* is smaller than threshold value. *Simulation* denotes results calculated by the parameter combination and *data* denotes experiment results. The computational code can be found in Code in File S1.

### Cellular automaton method

In cellular automaton method, cells are defined as agents with properties including division, transition and death. There are two kinds of agents: CSC and NSCC. NSCC can perform the behaviors including division, transition and death. CSC can execute symmetry and asymmetry divisions. The agents' behaviors are quantified by parameters which have been used in Equation (1). Each cell agent occupies a regular lattice with dimension of 10 µm×10 µm. In this model, 200×200 lattices were defined. A lattice is set to accommodate one cell at most at the same time. Therefore, a cell could divide into two cells unless there is at least one vacant site in its neighborhood (von Neumann neighborhood)[45].

## Supporting Information

Figure S1 Calculation of cell number of different initial conditions(t-log10(cell number)).
(TIF)

Figure S2 Parameters sensitivities of CSC proportion at equilibrium. Blue bars represent changes of CSC proportion at equilibrium when corresponding parameters are increased. Red bars represent changes of CSC proportion at equilibrium when corresponding parameters are decreased.
(TIF)

Figure S3 Calculation of cell number of different initial conditions under radiaion(t-log10(cell number)).
(TIF)

Figure S4 Phase portrait of analysis on equilibrium. R denotes the proportion of CSCs in the whole population. Solid circle stands for stable equilibrium, hollow circle stands for unstable equilibrium, and half-solid-half-hollow circle stands for half stable equilibrium.
(TIF)

File S1 Supporting code, discussion, equations, and tables. Table S1, Simulation of cell number. Table S2, Experiment data for Figure 2B: sorted NSCCs. Table S3, Experiment data for Figure 2B: sorted CSCs. Table S4, Simulation of cell number (Radiation).
(DOC)

Video S1 An example of CSCs' growth behavior with cellular automaton method. Red: CSC; Blue: NSCC; Black: vacant lattice.
(AVI)

Video S2 An example of NSCCs' growth behavior with cellular automaton method. Red: CSC; Blue: NSCC; Black: vacant lattice.
(AVI)
