# Supplementary figures and images for: Dynamics between Cancer Cell Subpopulations Reveals a Model Coordinating with Both Hierarchical and Stochastic Concepts
Source: PLoS One. 2014 Jan 9;9(1):e84654. doi: 10.1371/journal.pone.0084654 (PMC3886990; doi:10.1371/journal.pone.0084654)

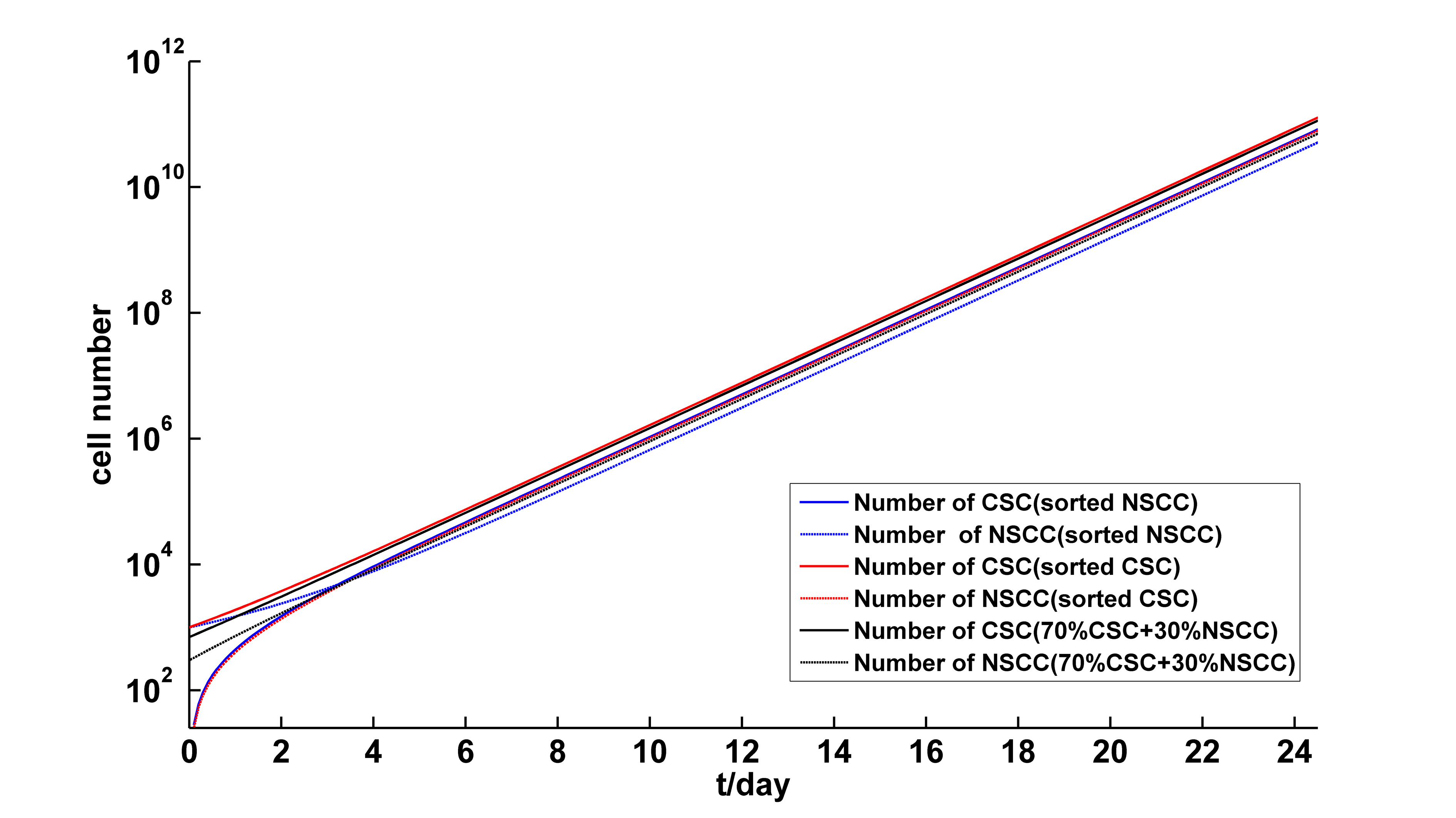

Supplement: Figure S1 — Calculation of cell number of different initial conditions(t-log10(cell number)). (TIF) [file pone.0084654.s001.tif]

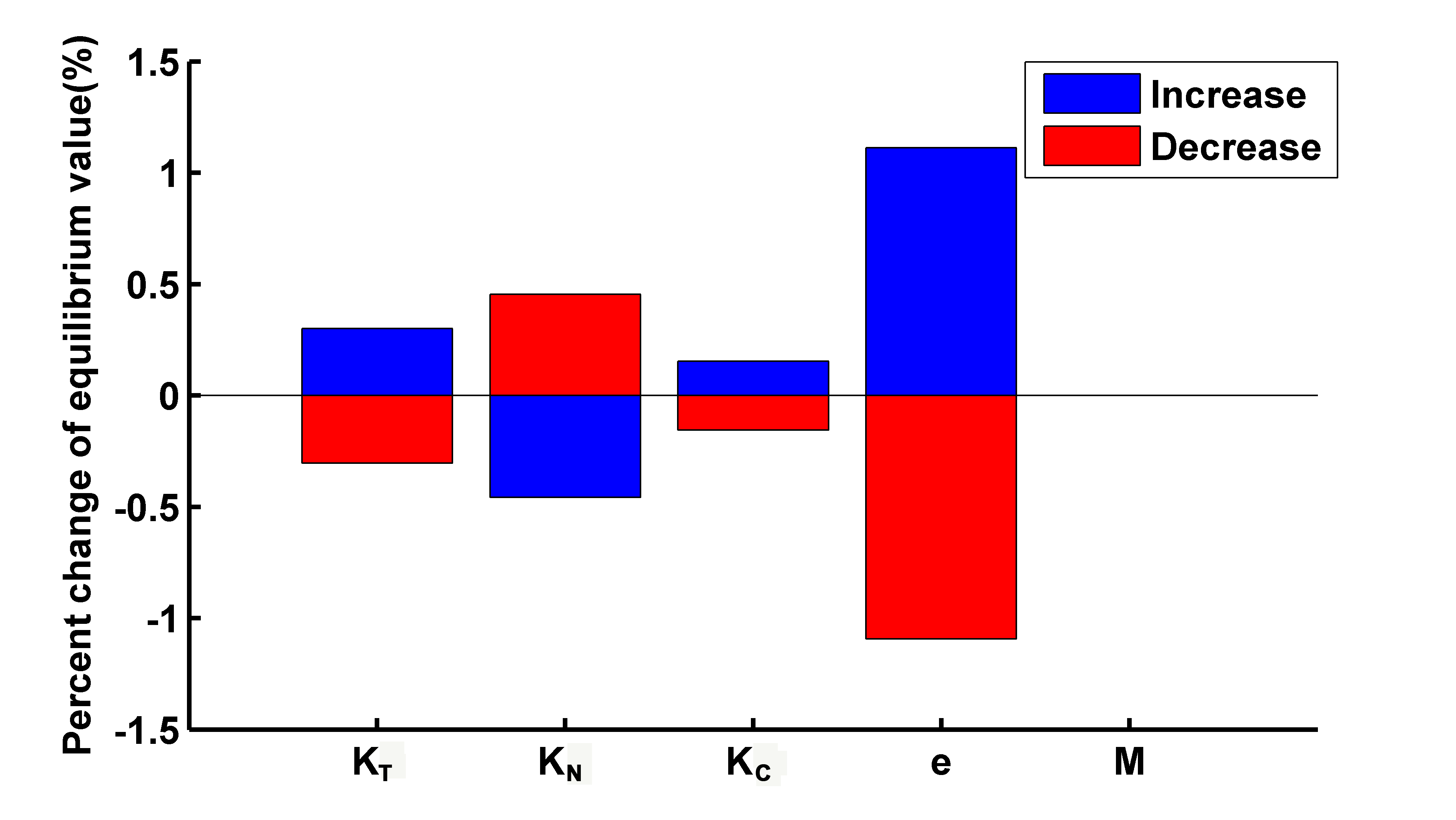

Supplement: Figure S2 — Parameters sensitivities of CSC proportion at equilibrium. Blue bars represent changes of CSC proportion at equilibrium when corresponding parameters are increased. Red bars represent changes of CSC proportion at equilibrium when corresponding parameters are decreased. (TIF) [file pone.0084654.s002.tif]

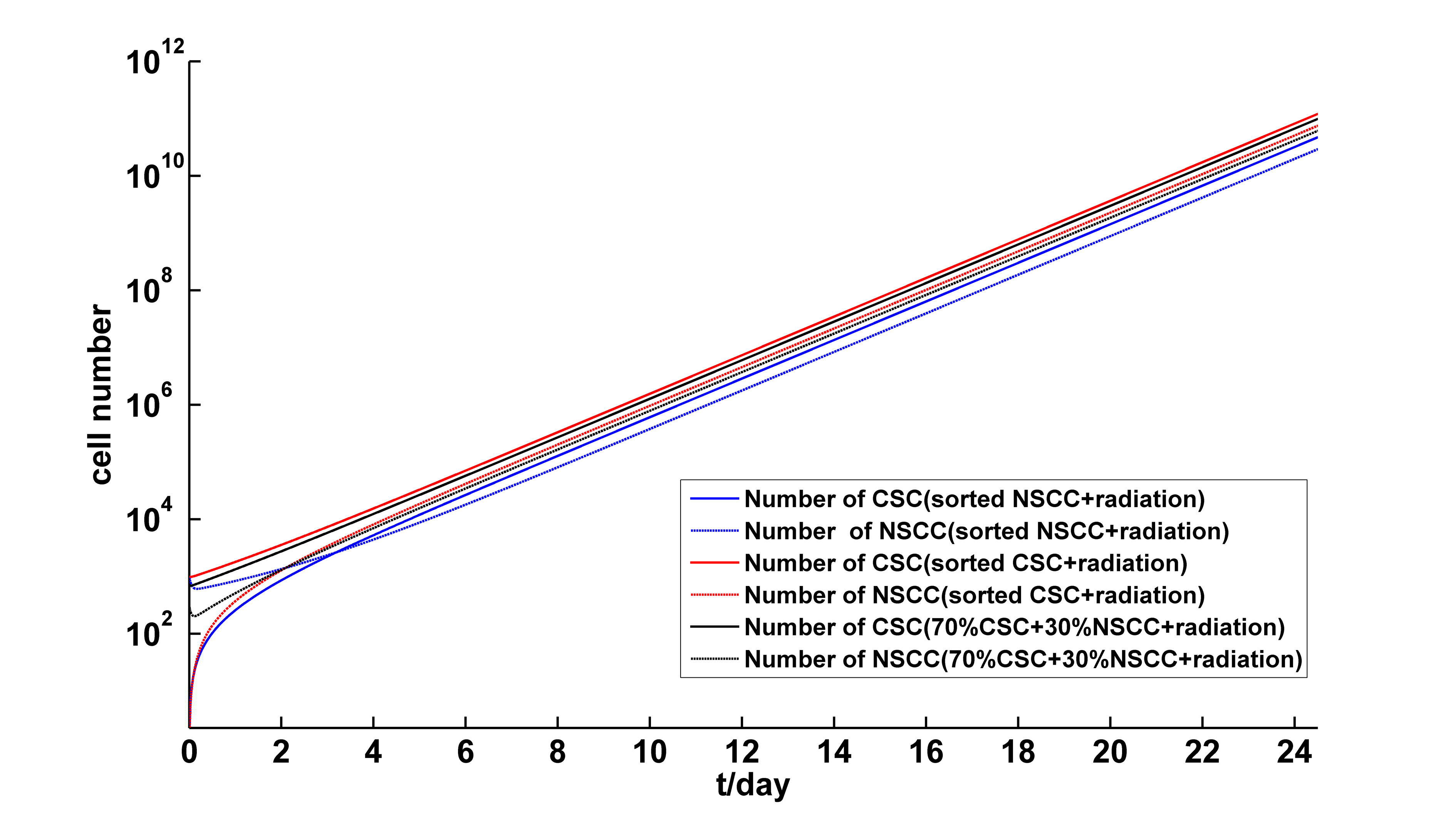

Supplement: Figure S3 — Calculation of cell number of different initial conditions under radiaion(t-log10(cell number)). (TIF) [file pone.0084654.s003.tif]
