# Supplementary material for: Dynamics between Cancer Cell Subpopulations Reveals a Model Coordinating with Both Hierarchical and Stochastic Concepts
Source: PLoS One. 2014 Jan 9;9(1):e84654. doi: 10.1371/journal.pone.0084654 (PMC3886990; doi:10.1371/journal.pone.0084654)
Supplement: File S1 — Supporting code, discussion, equations, and tables. Table S1, Simulation of cell number. Table S2, Experiment data for Figure 2B: sorted NSCCs. Table S3, Experiment data for Figure 2B: sorted CSCs. Table S4, Simulation of cell number (Radiation). (DOC) [file pone.0084654.s005.doc]

**Supplementary Information**

**Dynamics between cancer cell subpopulations reveals a model coordinating with both hierarchical and stochastic concepts**

Weikang Wang,^1^ Yi Quan,^1^ Qibin Fu,^1^ Yu Liu,^1^ Ying Liang,^1^ Jingwen Wu,^1^ Gen Yang,^1*^ Chunxiong Luo,^2^ Qi Ouyang,^2^ and Yugang Wang^1^

^1^ State Key Laboratory of Nuclear Physics and Technology, School of Physics, Peking University, Beijing 100871, P. R. China

^2^ Center for Microfluidic and Nanotechnology, The State Key Laboratory for Artificial Microstructures and Mesoscopic Physics, School of Physics, Peking University, Beijing 100871, P. R. China

**Supplementary Equations S1**

The production of DSBs can be described by a linear function of radiation dose .

$$B_{radiation}=B_{background}+kD$$

$B$denotes the average of DSBs per cell, $k$is the slope and$D$denotes dose.

The DSBs’ dynamics is described as following:

$$\frac{dB}{dt}=-rB$$

where$r$is repair rate of DSBs. So

$$B(t)=B_{0}\exp\left(-rt\right)$$

$B_{0}$is saturated number of DSBs and $B_{0}=B_{background}+kD$.

The death rate of cells treated by radiation is

$\frac{1}{2}m\left(B\left(t\right)\right)^{2}$.

Where $m$stands for lethal mis-repair rate of per DSB pair . Here, we neglect$B_{background}$.

The ODEs are

$$\begin{matrix} \frac{dC}{dt}=K_{T}\times\sum_{i=1}^{M-1} N_{i}+K_{C}\times e\times C-\frac{1}{2}m_{C}\left(kD\right)^{2}\exp(-2r_{C}t)\times C \\ \frac{dN_{1}}{dt}=-K_{N}\times N_{1}-K_{T}\times N_{1}+K_{C}\times\left(1-e\right)\times C-\frac{1}{2}m_{N}\left(kD\right)^{2}\exp(-2r_{N}t)\times N_{1} \\ ... \\ \frac{dN_{i}}{dt}=2\times K_{N}\times N_{i-1}-K_{N}\times N_{i}-K_{T}\times N_{i}-\frac{1}{2}m_{N}\left(kD\right)^{2}\exp(-2r_{N}t)\times N_{i} \\ ... \\ \frac{dN_{M}}{dt}=2\times K_{N}\times N_{M-1}-d\times N_{M} \end{matrix}$$

**Supplementary Equations S2**

In present study, to minimize the impact of transition rates on the growth rates, the growth rate *K_N_* and *K_C_* were calculated from the quantity changes of sorted CSCs and NSCCs in a short period (one day). Because CSCs and NSCCs’ cell cycles both are around one day, the division of newly born NSCCs in sorted CSCs population contribute little to quantity change in one day and the division of new CSCs in sorted NSCCs also can be treated as insignificant. We estimate *K_N_* and *K_T_* by

$$\begin{matrix} \frac{dN}{dt}\approx\left(K_{N}-K_{T}\right)\times N \\ \frac{dC}{dt}\approx K_{T}\times N \end{matrix}$$

The initial condition are sorted NSCCs.

And we estimate *K_C_* by

$$\begin{matrix} \frac{dC}{dt}\approx K_{C}\times e\times C \\ \frac{dN}{dt}\approx K_{C}\times\left(1-e\right)\times C \end{matrix}$$

The initial condition are sorted CSCs.

We could get the expression of *N* and *C* by using these equations and initial conditions. And then we infer these three parameters according to experiment data.

**Supplementary Discussion S1**

This model is simplified by introducing a constant death rate *K_D_* to replace age structure of NSCC.

The ODEs are as follows:

$$\begin{matrix} \frac{dN}{dt}=K_{C}\times\left(1-e\right)\times C+\left(K_{N}-K_{D}\right)\times N-K_{T}\times N \\ \frac{dC}{dt}=K_{C}\times e\times C+K_{T}\times N \end{matrix}$$

where $e=P_{S}-P_{D}$.

Define

$$K_{N}^{'}=K_{N}-K_{D}$$

Which is the net proliferation rate of NSCCs.

The equations become

$$\begin{matrix} \frac{dN}{dt}=K_{C}\times\left(1-e\right)\times C+\left(K_{N}^{'}-K_{T}\right)\times N \\ \frac{dC}{dt}=K_{C}\times e\times C+K_{T}\times N \end{matrix}$$

Define

$$R=\frac{C}{C+N}$$

*R* is the proportion of CSCs in the whole population.

We get

$$\frac{dR}{dt}=\left(K_{N}^{'}-K_{C}\right)\times R^{2}+\left(K_{C}\times e-K_{N}^{'}-K_{T}\right)\times R+K_{T}$$

The part on the right of equal mark is a quadratic equation.

Define

$$\begin{matrix} a=K_{N}^{'}-K_{C} \\ b=K_{C}\times e-K_{N}^{'}-K_{T} \end{matrix}$$

Now we explore the solutions of this quadratic equation when $\frac{dR}{dt}=0$. Which represent the proportion of CSCs at the equilibrium.

$$\frac{dR}{dt}=0\Rightarrow R=\frac{-\left(K_{C}\times e-K_{N}^{'}-K_{T}\right)\pm\sqrt{\left(K_{C}\times e-K_{N}^{'}-K_{T}\right)^{2}-4\times K_{T}\times\left(K_{N}^{'}-K_{C}\right)}}{2\times\left(K_{N}^{'}-K_{C}\right)}$$

1 Condition of $K_{T}>0$

(1) If$a>0$, so$b<0$

Since $-\frac{b}{a}>0$and$\frac{K_{T}}{a}>0$, there is at most one stable solution that satisfies$0\leq R\leq1$.

(2) If$a=0$, so$b<0$

There exists one stable solution.

$$R=\frac{K_{T}}{K_{N}^{'}+K_{T}-K_{C}\times e}$$

(3) If$a<0$, so$\frac{K_{T}}{a}<0$

There is at most one stable solution that satisfies$0\leq R\leq1$.

2 Condition of $K_{T}=0$

1. $a>0$, and the stable solution is$R=0$, and the other one is not a stable solution.
2. $a=0$, and the stable solution is$R=0$.

If $e=1$and initial condition is CSC ($R=1$), *R* will always be 1, but it is not the equilibrium)

$$a<0$$

2. If $b>0$, there exist at most one stable solution.

$$R=\frac{K_{C}\times e-K_{N}^{'}}{K_{C}-K_{N}^{'}}$$

The prerequisite for $0\leq R\leq1$ is$K_{C}\times e>K_{N}^{'}$. However, this requirement cannot be satisfied in our experiment or in most tumors .

(If *R* is initially zero, *R* will always be zero. But it is not stable. A little fluctuation will lead the ratio of CSC to$\frac{K_{C}\times e-K_{N}^{'}}{K_{C}-K_{N}^{'}}$. This situation, however, is impractical due to limited control over R)

1. If$b=0$, there is no stable solution in. But in this condition of$0\leq R\leq1$, $R=0$is a stable solution.
2. If$b<0$, the stable solution is$R=0$.

The phase portrait of analysis above is shown in Supplementary Fig. S3.

Furthermore, things will be a little different if NSCC has an age structure. Under the condition of *K_T_*=0, proportion of CSC is close to zero for most of the time. It is large enough to be detected by present experiment methods unless *e* is large or *M* is small enough as proportion of CSC $\propto\left(\frac{1+e}{2}\right)^{M}$.

**Supplementary Tables**

**Table S1. S**imulation of cell number

|  | sorted NSCCs | | sorted CSCs | | 70%CSC+30%NSCC | |
| --- | --- | --- | --- | --- | --- | --- |
| t/day | CSC | NSCC | CSC | NSCC | CSC | NSCC |
| 0 | 0 | 1.00E+03 | 1.00E+03 | 0 | 700 | 300 |
| 2 | 1.50E+03 | 2.39E+03 | 3.77E+03 | 1.37E+03 | 3.09E+03 | 1.68E+03 |
| 4 | 9.25E+03 | 7.80E+03 | 1.63E+04 | 8.60E+03 | 1.42E+04 | 8.36E+03 |
| 6 | 4.67E+04 | 3.17E+04 | 7.48E+04 | 4.40E+04 | 6.64E+04 | 4.03E+04 |
| 8 | 2.25E+05 | 1.42E+05 | 3.50E+05 | 2.13E+05 | 3.12E+05 | 1.92E+05 |
| 10 | 1.06E+06 | 6.63E+05 | 1.65E+06 | 1.01E+06 | 1.47E+06 | 9.09E+05 |
| 12 | 5.03E+06 | 3.12E+06 | 7.77E+06 | 4.80E+06 | 6.95E+06 | 4.30E+06 |
| 14 | 2.38E+07 | 1.47E+07 | 3.67E+07 | 2.27E+07 | 3.28E+07 | 2.03E+07 |
| 16 | 1.12E+08 | 6.95E+07 | 1.73E+08 | 1.07E+08 | 1.55E+08 | 9.60E+07 |
| 18 | 5.30E+08 | 3.28E+08 | 8.18E+08 | 5.07E+08 | 7.32E+08 | 4.54E+08 |
| 20 | 2.50E+09 | 1.55E+09 | 3.86E+09 | 2.39E+09 | 3.46E+09 | 2.14E+09 |
| 22 | 1.18E+10 | 7.33E+09 | 1.83E+10 | 1.13E+10 | 1.63E+10 | 1.01E+10 |
| 24 | 5.59E+10 | 3.46E+10 | 8.62E+10 | 5.34E+10 | 7.71E+10 | 4.78E+10 |

Table S2. Experiment data for Figure 2B: sorted NSCCs

| t/day | proportion of CSC(%) | error(%) |
| --- | --- | --- |
| 0 | 0.59 | 0.46 |
| 2 | 30.90 | 14.48 |
| 4 | 40.00 | 9.28 |
| 6 | 47.40 | 7.51 |
| 8 | 55.23 | 6.87 |
| 10 | 53.65 | 12.19 |
| 12 | 50.43 | 3.91 |
| 14 | 54.83 | 6.12 |
| 16 | 59.60 | 4.38 |
| 18 | 59.78 | 9.40 |
| 20 | 59.20 | 2.17 |
| 22 | 62.73 | 2.81 |
| 24 | 61.90 | 6.30 |

Table S3. Experiment data for Figure 2B: sorted CSCs

| t/day | proportion of CSC(%) | error(%) |
| --- | --- | --- |
| 0 | 99.43 | 0.45 |
| 2 | 83.50 | 0.50 |
| 4 | 78.68 | 1.90 |
| 6 | 73.23 | 9.46 |
| 8 | 71.50 | 2.40 |
| 10 | 69.40 | 9.91 |
| 12 | 70.55 | 5.28 |
| 14 | 71.40 | 5.26 |
| 16 | 71.35 | 5.59 |
| 18 | 73.45 | 3.08 |
| 20 | 71.23 | 6.66 |
| 22 | 67.88 | 5.61 |
| 24 | 65.67 | 5.87 |

**Table S4. S**imulation of cell number (Radiation)

|  | sorted NSCCs | | sorted CSCs | | 70%CSC+30%NSCC | |
| --- | --- | --- | --- | --- | --- | --- |
| t/day | CSC | NSCC | CSC | NSCC | CSC | NSCC |
| 0 | 0 | 1.00E+03 | 1.00E+03 | 0 | 700 | 300 |
| 2 | 8.61E+02 | 1.35E+03 | 3.58E+03 | 1.29E+03 | 2.76E+03 | 1.31E+03 |
| 4 | 5.28E+03 | 4.43E+03 | 1.55E+04 | 8.14E+03 | 1.24E+04 | 7.03E+03 |
| 6 | 2.66E+04 | 1.80E+04 | 7.09E+04 | 4.17E+04 | 5.76E+04 | 3.46E+04 |
| 8 | 1.28E+05 | 8.11E+04 | 3.31E+05 | 2.02E+05 | 2.70E+05 | 1.66E+05 |
| 10 | 6.07E+05 | 3.78E+05 | 1.56E+06 | 9.61E+05 | 1.27E+06 | 7.86E+05 |
| 12 | 2.87E+06 | 1.78E+06 | 7.36E+06 | 4.55E+06 | 6.01E+06 | 3.72E+06 |
| 14 | 1.35E+07 | 8.39E+06 | 3.47E+07 | 2.15E+07 | 2.84E+07 | 1.76E+07 |
| 16 | 6.40E+07 | 3.96E+07 | 1.64E+08 | 1.02E+08 | 1.34E+08 | 8.31E+07 |
| 18 | 3.02E+08 | 1.87E+08 | 7.75E+08 | 4.80E+08 | 6.33E+08 | 3.92E+08 |
| 20 | 1.43E+09 | 8.84E+08 | 3.66E+09 | 2.27E+09 | 2.99E+09 | 1.85E+09 |
| 22 | 6.74E+09 | 4.18E+09 | 1.73E+10 | 1.07E+10 | 1.41E+10 | 8.76E+09 |
| 24 | 3.18E+10 | 1.97E+10 | 8.17E+10 | 5.06E+10 | 6.68E+10 | 4.14E+10 |

**Supplementary Code: Code of simulated annealing algorithm**

*n=50*

*KN=zeros(n,1);*

*KC=zeros(n,1);*

*E=zeros(n,1);*

*seq=0;*

*while(seq<n)*

*Kn=0.5+0.5*rand(1);*

*Kc=0.5+0.5*rand(1);*

*e=rand(1);*

*T0=1e9;*

*Tmin=1e-10;*

*f=0.9;*

*T=T0;*

*inner=zeros(1,5);*

*tspan=0:2:24;*

*while (T>Tmin)*

*for innerlength=1:1:20*

*[t,y]=ode45(@sc,tspan,[B;sum(B(:))*49]);*

*total=zeros(length(tspan),1);*

*for i=1:1:length(tspan)*

*for j=1:1:M-1*

*total(i)=total(i)+y(i,j);*

*end*

*total(i)=total(i)+y(i,M+1);*

*end*

*ratio2=y(:,M+1)./total(:);*

*Eold=sum((result2-ratio2).^2);*

*Knold=Kn;*

*Kcold=Kc;*

*eold=e;*

*Knnew=Kn+(0.5*rand(1)-0.25)*(log10(T)+11)/20;*

*if(Knnew<0.5|Knnew>1)*

*Knnew=Knold;*

*end*

*Kcnew=Kc+(0.5*rand(1)-0.25)*(log10(T)+11)/20;*

*if(Kcnew<0.5|Kcnew>1)*

*Kcnew=Kcold;*

*end*

*enew=e+(1*rand(1)-0.5)*(log10(T)+11)/20;*

*if(enew<0|enew>1)*

*enew=eold;*

*end*

*Kn=Knnew;*

*Kc=Kcnew;*

*e=enew;*

*[t,y]=ode45(@sc,tspan,[B;sum(B(:))*49]);*

*total=zeros(length(tspan),1);*

*for i=1:1:length(tspan)*

*for j=1:1:M-1*

*total(i)=total(i)+y(i,j);*

*end*

*total(i)=total(i)+y(i,M+1);*

*end*

*ratio2=y(:,M+1)./total(:);*

*Enew=sum((result2-ratio2).^2);*

*if Enew<=0.05%*

*break;*

*end*

*if rand(1)<min(1,exp(-(Enew-Eold)/T))*

*Kn=Knnew;*

*Kc=Kcnew;*

*e=enew;*

*for i=1:1:4*

*inner(i)=inner(i+1);*

*end*

*inner(5)=Enew;*

*else*

*Kn=Knold;*

*Kc=Kcold;*

*e=eold;*

*end*

*if std(inner)<=0.01*

*break*

*end*

*end*

*if Enew<=0.05%*

*break;*

*end*

*T=f*T;*

*end*

**References**

1. Scott B (2011) Modeling DNA Double-Strand Break Repair Kinetics As An Epiregulated Cell-Community-Wide (Epicellcom) Response To Radiation Stress. Dose Response Volume 9, Number 4 / 2011: 579 - 601.

2. Sachs RK, Hlatky LR, Hahnfeldt P (2001) Simple ODE models of tumor growth and anti-angiogenic or radiation treatment. Mathematical and Computer Modelling 33: 1297-1305.

3. Li L, Neaves WB (2006) Normal stem cells and cancer stem cells: the niche matters. Cancer Res 66: 4553-4557.

4. La Porta CAM, Zapperi S, Sethna JP (2012) Senescent Cells in Growing Tumors: Population Dynamics and Cancer Stem Cells. PLoS Comput Biol 8: e1002316.
